# Changes in blood glucose and lipid metabolism levels in children with central precocious puberty and its correlation with obesity

**DOI:** 10.3389/fped.2024.1488522

**Published:** 2025-01-07

**Authors:** Xin Cui, Xin Sun, Qiubo Li, Zongbo Chen

**Affiliations:** ^1^ Department of Pediatrics, The Affiliated Hospital of Qingdao University, Qingdao, Shandong, China; ^2^ Department of Pediatrics, The Affiliated Hospital of Jining Medical University, Jining, Shandong, China; ^3^ Department of Encephalopathy, Integrative Medical Hospital of Jining City, Jining, Shandong, China

**Keywords:** precocious puberty, blood glucose, blood lipids, obesity, correlation

## Abstract

**Objective:**

This study analyzed the changes in blood glucose and lipid metabolism levels in children with central precocious puberty (CPP) and the correlation between CPP and obesity.

**Methods:**

In total, 88 children with CPP aged 6–10 years who were admitted to our hospital between January 2023 and June 2024 (the CPP group), and 88 children without CPP in the same age group who received health check-ups (the non-CPP group) were retrospectively enrolled in this study. General data [gender, age, bone age, and body mass index (BMI)] were collected. Levels of blood glucose metabolism indicators [fasting plasma glucose (FPG), 2-h postprandial blood glucose (2hPG), and hemoglobin A1c (HbA1c)] and blood lipid metabolism indicators [triglyceride (TG), total cholesterol (TC), low-density lipoprotein cholesterol (LDL-C), and high-density lipoprotein cholesterol (HDL-C)] were compared. The incidence of obesity was calculated, and the Tanner stages of the obese group and the non-obese group were compared. The correlation between CPP degree (measured by Tanner staging) and obesity degree (measured by BMI) was analyzed using Spearman’s correlation analysis.

**Results:**

The differences in gender and age between the CPP and non-CPP groups were insignificant (*P* > 0.05). Bone age and BMI in the CPP group were higher than in the non-CPP group (*P* < 0.05). The CPP group had higher serum FPG, 2hPG, HbA1c, TG, TC, and LDL-C levels and lower serum HDL-C levels than the non-CPP group. The incidence of obesity was higher in the CPP group (21.59%, 19/88) than in the non-CPP group (6.82%, 6/88). The Tanner staging scores in the obese group for the boys (testes and pubic hair), girls (breasts and pubic hair), and as a whole (testes/breasts and pubic hair) were elevated compared to those in the non-obese group (*P* < 0.05). Spearman’s correlation showed that the CPP degree (measured by Tanner staging) was positively correlated with the obesity degree (measured by BMI) in boys, girls, and the study sample as a whole (*P* < 0.001).

**Conclusion:**

Children with CPP had abnormal levels of blood glucose and lipid metabolism, and the CPP degree in these children was positively correlated with the degree of obesity.

## Introduction

Puberty refers to a complicated transitional phase in children that generally includes accelerated growth and the development of secondary sexual characteristics. It is a period of physical and psycho-social development (1). Central precocious puberty (CPP) is defined as the premature activation of the hypothalamic-pituitary-gonadal (HPG) axis, manifested by testicular enlargement in boys or breast development in girls earlier than the normal physiological age ranges (2). CPP classically occurs before the age of 8 years in girls and 9 years in boys (3). CPP is gonadotropin-dependent (4), and this condition can be diagnosed when the peak luteinizing hormone (LH) reaches ≥5.0 IU/L after being stimulated with gonadotropin-releasing hormones (5). The incidence of CPP is on the rise (6).

Children's bone age and other factors have been reported to have a close relationship with precocious puberty in children. It is helpful to remind parents to positively prevent these related factors and thereby prevent precocious puberty in children (7). Glucose is a crucial metabolic substrate for the production of tissue energy (8). Hemoglobin A1c (HbA1c), a glycated form of hemoglobin, can develop when glucose is elevated in the blood (9). Triglyceride (TG) in adipocytes has the ability to provide major metabolic energy stores in the body (10). Tanner staging can be applied to guide the differential diagnosis and aid in precocious puberty evaluation (11). A previous study reported that obesity is closely related to early sexual development, and earlier pubertal development has a positive association with obesity and central obesity in Chinese children (12). It has also been reported that there is a high prevalence of obesity has a high prevalence in girls with CPP at the time of diagnosis (13). Moreover, obesity is a commonly known risk factor for CPP (14). Girls diagnosed with CPP have adverse metabolic profiles at the time of diagnosis (15). Inspired by the research outlined above, our study analyzed and compared the changes in the levels of fasting plasma glucose (FPG), 2-h postprandial blood glucose (2hPG), HbA1c, TG, TC, low-density lipoprotein cholesterol (LDL-C), and high-density lipoprotein cholesterol (HDL-C) in children with CPP and those without CPP. Therefore, this study aimed to analyze the changes in blood glucose and lipid metabolism levels in children with CPP and the correlation of CPP with obesity.

## Materials and methods

### Ethics statement

The study was approved by the Ethics Committee of the Affiliated Hospital of Qingdao University (approval number: 20221011). The guardians of the participating children signed a written consent form.

### Study subjects and grouping

In total, 88 children with CPP aged 6–10 years who were admitted to the Affiliated Hospital of Qingdao University between January 2023 and June 2024 (the CPP group), and 88 children without CPP in the same age group who received health check-ups (the non-CPP group) were retrospectively enrolled. All the participating children were categorized into obese and non-obese groups based on the body mass index (BMI) classification criteria (Table 1).

**Table 1:** BMI classification criteria for excess weight screening in children aged 6–10 years (kg/m^2^).

Age (years)	Boys	Girls
∼6.0	17.7	17.5
∼6.5	18.1	18.0
∼7.0	18.7	18.5
∼7.5	19.2	19.0
∼8.0	19.7	19.4
∼8.5	20.3	19.9
∼9.0	20.8	20.4
∼9.5	21.4	21.0
∼10.0	21.9	21.5

#### CPP diagnostic criteria

(1) Girls who had developed secondary sexual characteristics before 7.5 years of age and had their first menstruation before 10 years of age; boys who had developed secondary sexual characteristics before 9 years of age. (2) Accelerated linear growth and increased annual growth rate compared to those of normal children. (3) Advanced bone age and bone age of more than the physiological age of 1 year. (4) Developed gonads and a pelvic B-ultrasound in girls showing increased uterine and ovarian volume and multiple follicles with a diameter ≥4 mm in the ovary; testicular volume ≥4 ml in boys. (5) The HPG axis function was initiated and serum gonadotropin and sex hormone levels reached pubertal levels.

### Diagnostic criteria for obesity in children aged 6–10 years

The cut-off points of the BMI of the participants were determined according to the research results of the National Health and Family Planning Commission of the People's Republic of China. The BMI calculation formula was: BMI = weight (kg)/height^2^ (m^2^). The detailed classification criteria are displayed in Table 1.

#### Inclusion criteria

(1) Boys aged 6–9 years old and girls aged 6–10 years old; (2) children with normal cognitive function who could understand and cooperate with the medical staff during sampling, examination for signs and symptoms, and other matters; who could comply medical procedures; and who could cooperate in the completion of the relevant examinations; (3) children without a history of taking sex hormones or weight-loss drugs; (4) children with complete clinical data.

#### Exclusion criteria

(1) Children with secondary CPP or obesity caused by drugs, diseases, or other reasons; (2) children with hyperthyroidism or hypothyroidism; (3) children with other endocrine diseases or infectious diseases; (4) children with central nervous system diseases or other serious systemic diseases; (5) children with malignant tumors.

#### Data collection and methods

(1) For the CPP and non-CPP groups, general data (gender, age, bone age, and BMI) were collected. The levels of blood glucose metabolism indicators (FPG, 2hPG, and HbA1c) and blood lipid metabolism indicators (TG, TC, LDL-C, and HDL-C) in the two groups were compared. The incidence of obesity in the two groups was calculated.

(2) For the obese and the non-obese groups, Tanner staging data were collected and compared. The staging of pubertal development (I–V) was carried out according to the Tanner staging criteria. Tanner stage II was considered a marker for the initiation of puberty. The relevant examination was conducted by specialized doctors. For boys, testes and pubic hair were examined for Tanner staging, and for girls, breasts, and pubic hair were examined. The detailed staging criteria are displayed in Table 2.

**Table 2:** Tanner staging criteria for children.

Tanner stage	Boys		Girls	
	Testes	Pubic hair	Breasts	Pubic hair
Stage Ⅰ	Toddler type, testicular volume 1–3 ml or diameter <2.5 cm	Prepubertal state, no pubic hair	Toddler type, undeveloped, flat breasts	Prepubertal state, no pubic hair
Stage Ⅱ	Bilateral testicular and scrotal enlargement, testicular volume 4–8 ml or diameter >2.5 cm, scrotal skin growing red and thin with wrinkles	A small amount of thin, straight pubic hair at the root of the penis, light in color, and distributed on the pubic bone and scrotum	Uplifted sporulated breasts, with the outer edge in or slightly beyond the areolae, slight tenderness in the glandular knot, uncolored areolae, and unenlarged nipples	Large labia with a small amount of thin, straight pubic hair that is light in color
Stage Ⅲ	Bilateral testes and scrotum enlargement, testicular volume 10–15 ml or diameter >3.5 cm	Deepened hair color; longer, thickened, and curled pubic hair extending upward to the pubic symphysis	Further enlarged breasts and areolae on the same mound. The nipples start to develop and protrude. The areolae begin to color	Deepened pubic hair color; thickened, longer, and curled pubic hair extending upward to the pubic symphysis
Stage Ⅳ	Scrotal skin color growing darker, with a volume of 15–20 ml or a diameter of approximately 4.0 cm	Increased amount of pubic hair; the pigmentation, thickness, and length of the hairs have the characteristics of adult pubic hair but do not reach the umbilicus and thigh	The breasts and areolae continue to increase, the areolae are obviously stained, and the sinuses are visible. The areolae form a second mound bulge on the breast, and the nipples increase significantly	The number of pubic hairs increases. The pigmentation, thickness, and length of the hairs have adult characteristics but are limited to the pubis
Stage Ⅴ	Adult type, testicular volume >20 ml or diameter >4 cm	Pubic hair continues to grow and thicken and extends to the medial thigh, thigh, and umbilicus, distributed in a diamond shape and reaching an adult type.	Complete development of adult-type breasts and the second mound of the areolae are disappearing	The characteristics and amount of pubic hair reach the adult level, located in the triangular distribution, and spread to the upper thigh

(3) Spearman’s correlation was used to analyze the correlation between CPP degree (measured by Tanner staging) and obesity degree (measured by BMI).

### Statistics

Statistical analysis was carried out using SPSS 26.0 software. Qualitative data were described as [*n* (%)] and the *χ*^2^ test was conducted. Quantitative data that were normally distributed were depicted as $\bar{x}\pm s$ and the independent samples *t*-test was performed; quantitative data that had a skewed distribution were depicted as M (P25, P75), and the Mann–Whitney *U* test was used. Correlation analysis was performed using Spearman’s correlation. *P* < 0.05 was indicative of a significant difference.

## Results

### General data

The differences in gender and age between the CPP and non-CPP groups were insignificant (*P* > 0.05). Bone age, bone age difference, and BMI in the CPP group were higher compared to those of the non-CPP group (*P* < 0.05) (Table 3).

**Table 3:** General data of the CPP and non-CPP groups.

Indicator	CPP group (*n* = 88)	Non-CPP group (*n* = 88)	*χ*2/Z	*P*
Gender (boy/girl, *n*)	32/56	38/50	0.854	0.355
Age (years)	8.00 (7.00, 9.00)	8.00 (7.00, 9.00)	−0.499	0.618
Bone age (years)	9.00 (9.00, 10.00)	8.00 (7.00, 9.00)	−7.556	<0.001
BMI (kg/m^2^)	19.43 (18.53, 20.38)	16.84 (15.90, 17.63)	−9.324	<0.001

### Blood glucose metabolism levels

The CPP group had higher serum FPG, 2hPG, and HbA1c levels compared to the non-CPP group (*P* < 0.05) (Table 4).

**Table 4:** Blood glucose metabolism levels in the CPP and non-CPP groups.

Indicator	CPP group (*n* = 88)	Non-CPP group (*n* = 88)	*Z*/*t*	*P*
FPG (mmol/L)	5.03 (4.56, 5.43)	4.23 (4.01, 4.45)	−8.093	<0.001
2hPG (mmol/L)	7.40 ± 0.43	6.97 ± 0.71	4.838	<0.001
HbA1c (%)	5.25 ± 0.48	5.01 ± 0.49	3.245	0.001

### Blood lipid metabolism levels

The CPP group had higher serum TG, TC, and LDL-C levels and lower serum HDL-C levels compared to the non-CPP group (*P* < 0.05) (Table 5).

**Table 5:** Blood lipid metabolism levels in the CPP and non-CPP groups.

Indicator	CPP group (*n* = 88)	Non-CPP group (*n* = 88)	Z	*P*
TG (mg/dl)	131.06 (99.95, 166.06)	89.31 (75.07, 103.23)	−7.976	<0.001
TC (mg/dl)	157.35 (139.10, 176.26)	150.12 (133.54, 166.07)	−2.167	0.030
LDL-C (mg/dl)	76.87 (63.87, 90.34)	66.07 (56.67, 75.65)	−4.833	<0.001
HDL-C (mg/dl)	49.16 (38.38, 60.81)	53.88 (45.67, 62.21)	−2.148	0.032

### Tanner staging in children with obesity

The incidence of obesity was elevated in the CPP group (21.59%, 19/88) compared to the non-CPP group (6.82%, 6/88) (*P* < 0.05). The Tanner staging scores for the boys (testes and pubic hair), girls (breasts and pubic hair), and as a whole (testes/breasts and pubic hair) were higher in the obese group compared with the non-obese group (*P* < 0.05) (Table 6).

**Table 6:** Tanner stages in the obese and non-obese groups (points).

Gender	Obese group (*n* = 25)	Non-obese group (*n* = 151)	*Z*	*P*
Boys (*n* = 70)				
Testes	2.00 (1.00, 2.00)	0.00 (0.00, 2.00)	−3.129	0.002
Pubic hair	2.00 (1.00, 2.00)	1.00 (0.00, 2.00)	−2.690	0.007
Girls (*n* = 106)				
Breasts	2.00 (2.00, 2.75)	1.00 (0.00, 2.00)	−2.906	0.004
Pubic hair	2.00 (2.00, 2.00)	1.00 (0.00, 2.00)	−2.403	0.016
Total number (*n* = 176)				
Testes/breasts	2.00 (1.50, 2.00)	1.00 (0.00, 2.00)	−4.044	<0.001
Pubic hair	2.00 (1.50, 2.00)	1.00 (0.00, 2.00)	−3.376	0.001

### Correlation analysis of the CPP degree and obesity degree

Spearman’s correlation showed that the CPP degree (measured by Tanner staging) had a positive correlation with obesity degree (measured by BMI) in boys, girls, and as a whole (*P* < 0.001) (Table 7).

**Table 7:** Correlation analysis of Tanner stages and BMI.

Indicator		Boys		Girls		Whole	
		Testes	Pubic hair	Breasts	Pubic hair	Testes/breasts	Pubic hair
BMI	*r*	0.818	0.789	0.721	0.728	0.778	0.769
	*P*	<0.001	<0.001	<0.001	<0.001	<0.001	<0.001

## Discussion

CPP, the premature activation of the HPG axis, results in the onset of sexual development in girls and boys at a young age (16). The number of children with precocious puberty is increasing globally. Precocious puberty may exert negative effects on both the physical and mental health of children (17). This article aimed to analyze changes in blood glucose and lipid metabolism levels in children with CPP and the correlation of CPP with obesity.

In our article, we collected general data from children with CPP and children without CPP, and, after analyzing and comparing these data, we found that the bone age difference and BMI of the CPP group were higher compared with the children without CPP. It has been reported that girls undergoing puberty have higher bone age (18). Girls with CPP have also been reported to have higher fasting insulin, TG, and LDL-C levels at the time of diagnosis (15). In addition, lipid profile contents have been demonstrated to be altered in girls with precocious puberty compared with healthy controls. TG, TC, and low-density lipoprotein levels were elevated in girls with precocious puberty (19). In our study, we observed the changes in blood glucose and lipid metabolism indicator levels between the CPP and non-CPP groups and found that the CPP group had higher serum FPG, 2hPG, and HbA1c levels; higher serum TG, TC, and LDL-C levels; and lower serum HDL-C levels compared with the non-CPP group. Further, we grouped the study participants into an obese group and a non-obese group and compared the Tanner stages of the participants in these two groups. It was observed that the incidence of obesity was raised in the CPP group (21.59%) compared with the non-CPP group (6.82%). The Tanner staging scores for the boys (testes and pubic hair), girls (breasts and pubic hair), and as a whole (testes/breasts and pubic hair) were higher in the obese group than in the non-obese group. In addition, we analyzed the correlation of CPP degree with obesity degree, and Spearman’s correlation showed that the CPP degree (measured by Tanner staging) had a positive relationship with obesity degree (measured by BMI) in boys, girls, and as a whole. As previously reported, there is a confirmed relationship between obesity and CPP (20). Furthermore, obesity is a key underlying cause of CPP, and prolonged obesity in early childhood might act as a risk factor for CPP, particularly in girls (21). Moreover, CPP may also be considered a risk factor for metabolic disorders (22). Obese children have been observed to enter puberty at an earlier age compared with non-obese children. In girls with CPP, it has been demonstrated that an increased BMI is associated with slightly lower peak-stimulated LH levels in Tanner stages 2 and 3 (23). Taken together, children with CPP exhibit abnormal glucose and lipid metabolism levels, and the severity of CPP in children is correlated with the degree of obesity.

It is commonly accepted that nutrition is one of the most significant factors influencing the timing of growth and the transition to maturity. During development, the intake of nutrients promotes signaling through insulin-like systems that govern the growth of cells and tissues and, furthermore, it regulates the timely production of the steroid hormones that initiate the juvenile-adult transition (24). It has been reported that a high-fat diet is associated with alterations in biochemical and neuroendocrine pathways and a pro-inflammatory state. It may contribute to the activation of the hypothalamic-pituitary-gonadal axis. Enhancing our understanding of the effects of a high-fluoride, low-fat diet may aid in developing strategies to prevent precocious puberty in obese children. Promoting behaviors that avoid the consumption of high-fat diets may help protect children's physiological development and reproductive health (25). A study also indicated that prolonged overweight and obesity in early childhood may be risk factors for CPP, especially in girls. Weight loss may be an important approach for the prevention of precocious puberty in children (21). Considering the previously mentioned abnormal lipid metabolism in the CPP group, excessive fat accumulation in obese children may lead to a significant increase in estrogen levels, accelerating the development of secondary sexual characteristics in these children. This disruption of the endocrine system may further impact lipid metabolism, resulting in changes in the lipoprotein profile. In addition, excessive intake of high-calorie and high-fat foods is a significant contributor to obesity. These environmental factors not only contribute to obesity but may also further influence lipid metabolism and sexual development by affecting the endocrine system. However, the underlying mechanisms causing significant differences in lipoprotein profiles between patients with precocious puberty and other groups may involve multiple aspects, including the aforementioned factors, genetics, and lifestyle habits. Further in-depth research should be conducted to explore these mechanisms.

In conclusion, this research found that children with CPP had abnormal levels of blood glucose and lipid metabolism, and the CPP degree in children had a positive correlation with the obesity degree. This study lays a foundation by examining the changes in blood glucose and lipid metabolism indicator levels in children with CPP and analyzing the correlation between CPP degree and obesity degree. Our study is based on limited clinical data, and, therefore, our study results are only representative of a small group of children with CPP. Furthermore, previous studies have suggested that enhancing our understanding of the impact of a high-fluoride, low-fat diet may aid in developing strategies to prevent precocious puberty in obese children. However, this study did not conduct a detailed stratified analysis based on diet. Future research should further explore the changes in blood glucose and lipid metabolism levels in children receiving different diets and their relationship with precocious puberty. This would provide a scientific basis for formulating targeted prevention and treatment strategies.

## Data Availability

The original contributions presented in the study are included in the article/Supplementary Material, further inquiries can be directed to the corresponding author.
